# Convolutional Neural Network-Based Humerus Segmentation and Application to Bone Mineral Density Estimation from Chest X-ray Images of Critical Infants

**DOI:** 10.3390/diagnostics10121028

**Published:** 2020-11-30

**Authors:** Yung-Chun Liu, Yung-Chieh Lin, Pei-Yin Tsai, Osuke Iwata, Chuew-Chuen Chuang, Yu-Han Huang, Yi-Shan Tsai, Yung-Nien Sun

**Affiliations:** 1 Department of Biomedical Engineering, Da-Yeh University, Changhua 51591, Taiwan; yungchun7@gmail.com; 2 Department of Pediatrics, National Cheng Kung University Hospital, College of Medicine, National Cheng-Kung University, Tainan 70457, Taiwan; drapple@mail.ncku.edu.tw; 3 Department of Obstetrics and Gynecology, National Cheng Kung University Hospital, College of Medicine, National Cheng-Kung University, Tainan 70457, Taiwan; tsaipy@mail.ncku.edu.tw; 4 Department of Neonatology and Pediatrics, Nagoya City University Graduate School of Medical Science, Nagoya, Aichi 467-8601, Japan; o.iwata@med.nagoya-cu.ac.jp; 5 Department of Computer Science & Information Engineering, National Cheng Kung University, Tainan 701, Taiwan; micky80828@gmail.com (C.-C.C.); s946190@gmail.com (Y.-H.H.); 6 Department of Medical Imaging, National Cheng Kung University Hospital, College of Medicine, National Cheng-Kung University, Tainan 70457, Taiwan; 7 Clinical Innovation and Research Center, National Cheng Kung University Hospital, College of Medicine, National Cheng-Kung University, Tainan 70457, Taiwan; 8 AI Biomedical Research Center, Ministry of Science and Technology, Tainan 701, Taiwan

**Keywords:** X-ray segmentation, deep learning, premature infants, bone mineral density, rickets, osteopenia of prematurity

## Abstract

Measuring bone mineral density (BMD) is important for surveying osteopenia in premature infants. However, the clinical availability of dual-energy X-ray absorptiometry (DEXA) for standard BMD measurement is very limited, and it is not a practical technique for critically premature infants. Developing alternative approaches for DEXA might improve clinical care for bone health. This study aimed to measure the BMD of premature infants via routine chest X-rays in the intensive care unit. A convolutional neural network (CNN) for humeral segmentation and quantification of BMD with calibration phantoms (QRM-DEXA) and soft tissue correction were developed. There were 210 X-rays of premature infants evaluated by this system, with an average Dice similarity coefficient value of 97.81% for humeral segmentation. The estimated humerus BMDs (g/cm^3^; mean ± standard) were 0.32 ± 0.06, 0.37 ± 0.06, and 0.32 ± 0.09, respectively, for the upper, middle, and bottom parts of the left humerus for the enrolled infants. To our knowledge, this is the first pilot study to apply a CNN model to humerus segmentation and to measure BMD in preterm infants. These preliminary results may accelerate the progress of BMD research in critical medicine and assist with nutritional care in premature infants.

## 1. Introduction

Rickets of prematurity, also known as osteopenia of prematurity, is a major problem and is common among premature infants [1]. The decrease in the amount of calcium and phosphorus in the bones causes them to be weak and brittle and increases the risk of broken bones. Mobilization of patients to the image laboratory, however, for quantification of bone density becomes risky because of critical clinical scenarios. Hence, rickets of prematurity becomes difficult to diagnose and often is neglected. Diagnosis is often delayed until the patients have suffered pain from humeral or femoral fractures. Several biomarkers are related to osteopenia of prematurity [2], such as calcium, phosphate, and alkaline phosphatase. However, the bone density may not be well correlated with any single biomarker [3,4]. Even with a fracture, these biomarkers still do not present a good corresponding response.

The current gold standard for the quantification of bone mineral density (BMD) is dual-energy X-ray absorptiometry [5,6], also known as DEXA or DXA. The clinical availability of DEXA is very limited in radiology departments, although it is a simple and rapid diagnostic procedure. DEXA is often unavailable for critical patients who stay in intensive care units, such as preterm infants or other adult patients. Life-support tubing, thermal control, and respiratory support in critical preterm infants is difficult for BMD study with DEXA. Alternative methods of DEXA, such as bedside ultrasound studies, are reported but rely on examiner experience and expensive ultrasound equipment [7].

**Figure 1:**
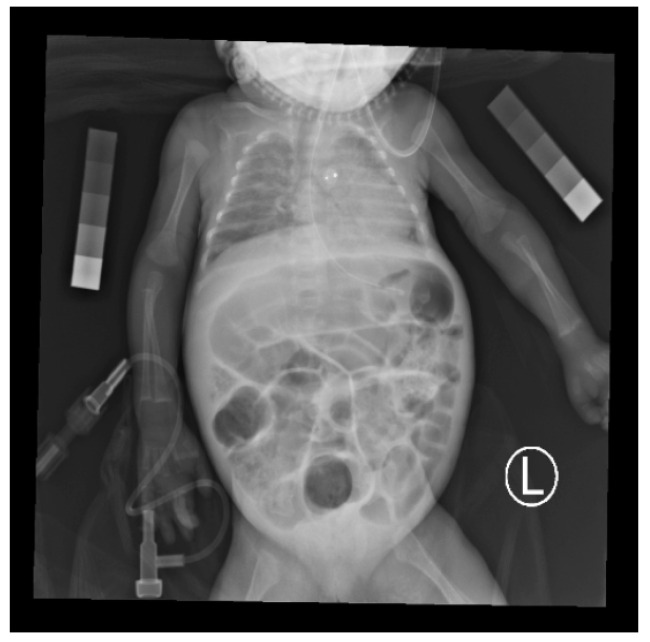
A chest X-ray image of a premature infant with standardized density phantoms.

A simple X-ray image was proposed as an alternative for DEXA. In the literature, X-ray of the hand bone has been suggested for bone density measurement [8,9]. The measurement in Sotoca’s study [8] could have a good correlation with the true bone density obtained by DEXA (with a correlation coefficient value of 0.79). Extra radiation exposure, however, was needed for immature preterm infants. Premature infants often require chest X-rays (CXR) for respiratory condition studies in neonatal intensive care units (NICU). Hence, physicians may be able to use the X-ray to identify rickets by examining areas such as the humerus, ribs, and thoracic vertebrae (Figure 1). To use routine CXR for bone density analysis has seldom been discussed, but we consider its potential feasibility for vulnerable preterm infants.

To diagnose and quantify bone density accurately, however, depends on the physician’s experience, which makes computer-assisted reading a necessity. In this study, humerus bone images with a standardized density of phantoms on routine chest radiographs of premature infants were obtained for BMD calculation (Figure 1). This study planned to develop a well-developed computer-aided analysis tool with a standardized density of phantoms that may be helpful to achieve a quantitative estimation of BMD during routine care without mobilizing critical patients.

To make good segmentation of selected bones from X-ray images is an important first step for the further accurate calculation of bone density. Sotoca et al. [8] developed an accurate and reliable X-ray system for bone mass assessment, in which an active shape model algorithm was used to segment the hand bone. Zhang et al. [10] proposed a method for osteoporosis grading, whereby a regional growth model, a dynamic contour model, and the Otsu method were applied to segment the humerus. Sapthagirivasan and Anburajan [11] presented an osteoporotic risk detection system using a support vector machine classifier for digital hip radiographs, in which the region of interest (ROI) was placed and manually cropped on the neck region of each radiograph.

Although the accuracy of the conventional segmentation was satisfactory, most of the studies depended on human labor, manual initialization, and image preprocessing. Figure 1 shows a chest X-ray image of a premature infant. It can be observed that the image is complex, and automatic segmenting of the humerus from the image would be difficult. Computer-assisted segmentation might be helpful for clinicians in this situation.

Deep learning convolutional networks have achieved high accuracy in medical image segmentation in recent years [12,13]. The application of a convolutional neural network (CNN) to image segmentation can be roughly divided into two categories. The first uses the pixel classification results as the output. Such a method uses many small patches to train the network model and then uses the network to identify the class of each pixel in the image. For example, Arevalo et al. [14] proposed the use of a convolutional neural network for basal cell carcinoma microscopic images, in which the normal area is represented in blue and the cancer cell area in red. Xu et al. [15] used a convolutional neural network to segment brain tumors on microscopic images. Ibragimov and Xing [16] adopted 13 convolutional neural networks to segment different organs at risk from head and neck CT images. Cernazanu-Glavan and Holban [17] proposed a CNN-based segmentation method of the bone structure from X-ray images. The segmentation method in these studies, however, would need more computation time and was unable to learn global features. The second category uses the entire image of the dataset as the input to train the segmentation model. The training and testing do not need any image preprocessing or ROI initialization, and the segmentation is operated end-to-end. For example, Wang et al. [18] proposed a convolutional neural network using an encoding and decoding architecture to segment the wound region on a color image and analyzed the segmented area to estimate the wound healing status of patients in different periods. Arif et al. [19] presented a localization framework of the cervical spine. Three segmentation CNNs were investigated to segment the cervical spine on X-ray images. Tong et al. [20] proposed fully convolutional neural networks with a shape representation model to segment organs at risk on head and neck CT images. Zabihollahy et al. [21] segmented the whole gland, central gland, and peripheral zone of the prostate from T2-weighted and apparent diffusion coefficient map prostate MR images by using U-nets. These CNNs contained deconvolution or up-sampling layers so that the output of the network was the segmentation result of the whole image directly. The CNN structures used in the second category make the segmentation convenient. An end-to-end CNN segmentation method was applied in this study. An X-ray image is sent to a segmentation CNN without image preprocessing and the output is the segmentation result of the humerus. Generally, the segmentation performance improves when increasing the depth of the network, but it will suffer from a huge number of parameters. A fully convolutional DenseNet (FC-DenseNet) [22] was designed that extended the usage of DenseNet [23] to segmentation. This structure showed efficient use of multilevel feature maps and good segmentation performance with a large reduction in parameter usage.

This study proposes a CNN-based humerus segmentation system from premature infant X-ray images by using FC-DenseNet. To our knowledge, this is the first pilot study to apply a CNN model to the humerus segmentation in preterm infants. It is hoped that the preliminary results of this study will accelerate the progress of related research and assist the care of premature infants. Following early and accurate diagnosis for bone problems, physicians can prevent and even treat early the metabolic bone disease of premature infants.

## 2. Materials and Methods

### 2.1. Study Design and Settings

The prospective study protocol was approved by the institutional review board of NCKUH (A-ER-105-271, December 1^st^ 2016). This study was conducted at a 20-bed tertiary neonatal intensive care unit (NICU) at the National Cheng Kung University Hospital in Tainan, Taiwan. Signed informed consent was obtained from all parents prior to enrollment of their infants in the study.

Routine chest radiographic images of premature infants and newborn babies were obtained by the clinical indication. During preparation for the imaging procedure, two commercial QRM-DEXA calibration phantoms were placed separately near the upper arms of the infant by nurses.

The base material of the QRM-DEXA phantom was calcium hydroxyapatite of appropriate grain size. The bone equivalent inserts had a specified bone mineral density of 50, 100, 200, 400, and 800 mg HA/cm^2^. The phantom was used as a calibration device in the radiograph to determine the relationship between bone density and gray level and normalize radiographs between different patients.

The X-ray images were acquired from the portable digital radiography system. Technical factors were tube voltage with fixed 50 kV, exposure condition 0.8 mAs, source-film distance 60 cm, and exposure index 1400–1800. The field of view in each chest X-ray image contained the whole chest and both arms with forearm supination.

### 2.2. Bone Segmentation

**Figure 2:**
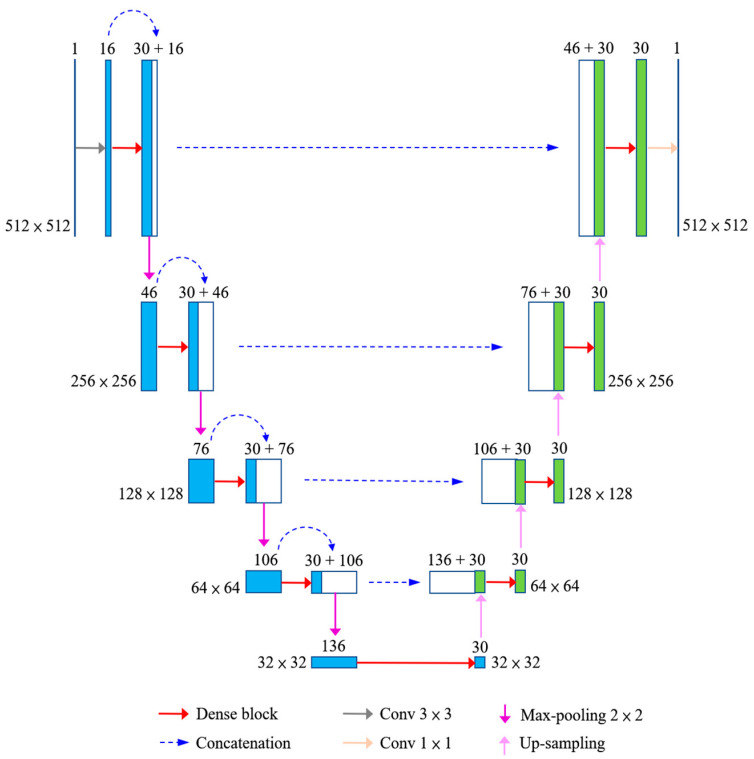
Network architecture of the proposed system.

This study proposed a CNN-based segmentation system for the humerus on X-ray images by using FC-DenseNet. The network architecture is shown in Figure 2. The gray level image was sent to a convolutional layer with a 3 × 3 filter size and a feature map with the output channel number set to 16. This feature map was sent to a dense block, and the output was concatenated with its input to form a concatenated feature map. The feature map was down-sampled by performing max pooling with a size of 2 × 2. The down-sampled feature map was sent to a dense block again. These steps were repeated four times on the down-sampling side. The last down-sampled feature map was sent to a dense block directly. The output was up-sampled, and the result was concatenated with the corresponding feature map from the down-sampling side. The concatenated feature map was sent to a dense block. These steps were repeated four times on the up-sampling side. The last dense block output is sent to a convolutional layer with a 1 × 1 filter size, and the segmentation result is generated.

**Figure 3:**
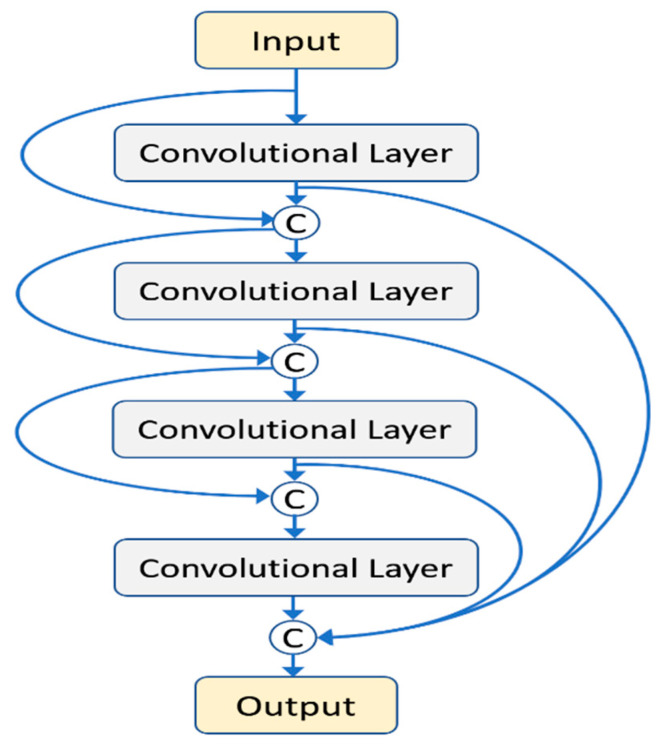
A dense block of four layers.

A dense block is shown in Figure 3, which is the key component of the FC-DenseNet. The example shows a dense block with four layers (*l* = 4), and the growth rate equals 3 (*r* = 3). The output of each convolutional layer would concatenate with its input to form an input to the next layer inside the block. A circle with a symbol C represented the concatenation of feature maps. The growth rate r denoted the channel number of the output feature maps for each convolutional layer. The output of a dense block was the concatenation of the outputs of all previous layers and thus contained 12 feature maps. This design allowed the multilayer feature maps to be used efficiently. The layer was set to 5 (*l* = 5), and the growth rate was set to 6 (*r* = 6) for the dense block in this study.

There were 210 routine chest X-ray images of premature infants used in this study. These images were grayscale and approximately 2000 × 1500 or 1500 × 1500 pixels in size. For each image, segments of the left and right humerus of the infant were annotated by an expert as the ground truth. The images were warped to the size of 512 × 512 in the system. A five-fold cross-validation process was employed. The dataset was randomly divided into five groups. Each of the five groups alternately served as the unseen testing dataset, while the remaining four groups merged as the training dataset. Augmentation of rotation with ±15 degrees was applied for the training dataset. The augmented training dataset contained 504 images in the five-fold cross-validation process. No pre-processing was applied, and the images were trained and tested end-to-end. The testing results from the five groups were summed up to obtain the testing statistics of a five-fold test instance.

In the training phase, the CNN network parameters were randomly initialized, the batch size was set to 4, and the learning rate was set to 0.001 with a decay of 0.5% after each epoch. An Adam optimizer was applied to train the network, and the L2-norm loss function was used for the optimization. The L2 loss function was calculated by the mean square error between the ground truth and the predicted result, with the equation shown below:

(1) $$L2\;loss=\frac{\sum_{i=1}^{N}{\left(y_{i}-p\left(x_{i}\right)\right)}^{2}}{N}$$

where *x_i_* is the input data, *y_i_* is the ground truth, *N* is the total number of data points, and *p*(*x_i_*) is the predicted result. The training stops after 100 epochs, and training time was about 6.8 h. This study found that this proposed system converges after about 100 epochs, and the training procedure was stopped at this number to avoid overfitting. In the testing phase, the image was sent to the network, and the output was the segmentation results of the left and right humerus. The system was run on a PC with Intel Core i7 3.60 GHz CPU, 16G memory, and NVIDIA GeForce GTX 1080Ti GPU. The network was implemented based on the TensorFlow framework in Python. The parameter settings of the proposed network are summarized as the following Appendix A Table A1.

### 2.3. Association between DEXA Phantom Brightness and BMD Value

**Figure 4:**
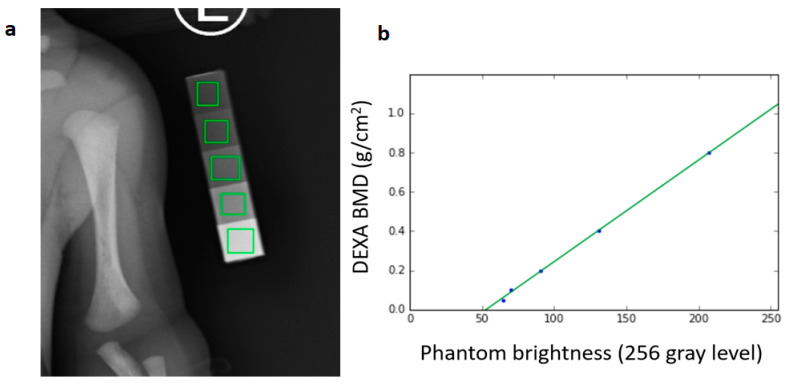
(**a**) A five-step phantom in the radiograph. Five green squares are manually selected to measure the average brightness of each step region. (**b**) The relationship between phantom brightness and corresponding dual-energy X-ray absorptiometry bone mineral density (DEXA BMD).

Evaluating rickets (osteopenia) of prematurity is essential for nutritional management. However, a well-defined and consistently quantifiable method to monitor rickets of prematurity is not presently available in the standard clinical setting. An alternative approach to estimating BMD from routine chest X-rays of premature infants is suggested in this study. BMD in preterm infants could thus be estimated in a non-interfering manner. Prior to BMD estimation, the relationship between DEXA phantom brightness and BMD value (the ground truth) needed to be investigated. This relationship was formulated in the following steps. A standard digital X-ray of the chest exposed along with a DEXA calibration phantom was obtained. There were five-step regions on the phantom. In each step region, a square in each region was selected manually, as shown in Figure 4a, to measure the average brightness of the step region. The five measured average brightness values and their corresponding known BMD values of the step regions were used to plot the calibration diagram, as shown in Figure 4b. A linear regression algorithm [24] was used to find out the calibration relationship, and a linear regression equation LRE for the X-ray image would be obtained. The results showed that the correlation coefficient between these two kinds of values is 0.9996. Then the analysis could use LRE to estimate the BMD of the bone.

### 2.4. BMD Estimation in Prematurity

**Figure 5:**
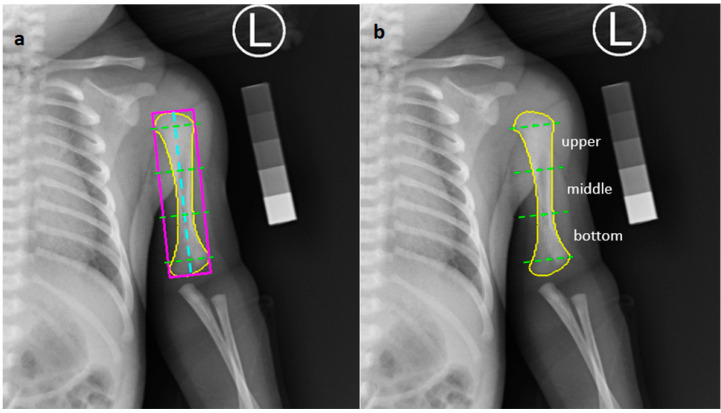
(**a**) The yellow contour is a segmented area, the pink rectangle is the minimum-bounding rectangle of the segmented area, and the blue dashed line is the central axis of the humerus. (**b**) Three separate areas of the bone are determined for further BMD estimation.

The results of the humerus segmentation were used to further estimate BMD. Given a segmented area, such as the yellow contour in Figure 5a, we tried to obtain the minimum-bounding rectangle (MBR) that contains the area. The method proposed by Freeman et al. [25] was adopted to determine the minimum-area encasing rectangle of the segmented bone area, such as the pink rectangle in Figure 5a. Next, the information of MBR was used to decide the center axis of the humerus. The axis that passes through the center of MBR and parallels to the longer side of the MBR was regarded as the central axis; see the blue dashed line in Figure 5a. Then, the central axis was divided into 11 equivalent segments. Excluding the top and bottom sections, the rest was separated into three equivalent parts; see the green dashed lines in Figure 5a. Three separate areas of bone were then determined for further BMD estimation, denoted as the upper region, the middle region, and the bottom region, as shown in Figure 5b. The average brightness of each region was calculated, and then the estimated BMD for each region was calculated by the regression equation LRE.

**Figure 6:**
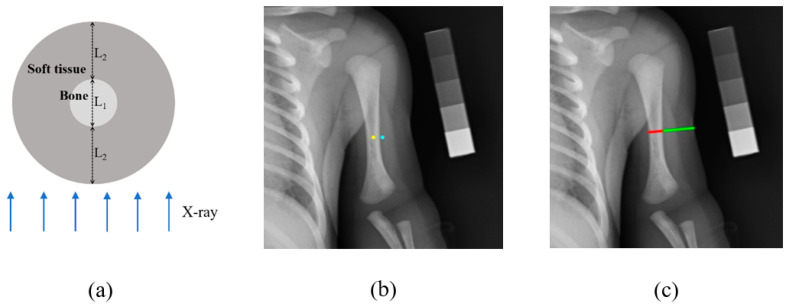
(**a**) The transverse view of the premature infant’s upper arm. (**b**) The yellow dot is a representative point of the bone area to be estimated, while the blue dot is a representative point of the soft tissue close to the bone. (**c**) Line segments are drawn on the X-ray image by an expert.

In an X-ray image, the bone and soft tissue can be observed simultaneously. Since the bone is covered by soft tissue, as illustrated in Figure 6a, the imaging of the bone area is affected by soft tissue. This effect must be eliminated to obtain more accurate bone brightness and bone density assessment. A soft tissue correction method was proposed to solve the abovementioned problem and estimate bone mineral density using the following equation:

(2) $$BMD_{estimated\;}=\;T\left(I_{y}\right)-\;T\left(I_{b}\right)*S$$

where *I_y_* is the average brightness of the bone area to be estimated, and *I_b_* is the average brightness of pixels (15 outside the left and right boundary lines of the bone area to be estimated). The yellow dot in Figure 6b was taken as a representative point of the bone area to be estimated, and the blue dot was taken as a representative point of the soft tissue close to the bone. *T* is the transfer function related to LRE. *S* is the adjustment parameter, which is obtained by the equation below:

(3) $$S=\frac{2L_{2}}{L_{1}+2L_{2}}$$

where *L*_1_ and *L*_2_ represent the length of the red and the green line segments shown in Figure 6c. Since the portion of the middle section of the premature infant’s upper arm was approximately cylindrical, as illustrated in Figure 6a, the thickness of the soft tissue was approximately 2 × *L*_2_. *S* is close to the ratio of the thickness of the soft tissue to the thickness of the arm. As shown in Figure 6c, the red and green line segments were drawn on the middle section of the upper arm bone on the X-ray image by an expert. The expert marked the image through the computer program and the length of the segments are calculated automatically.

## 3. Results

### 3.1. Qualitative Evaluation of the Humerus Segmentation

In addition to the proposed system, two other segmentation CNNs, U-net [26] and auto-encoder (AE) were built for comparison. U-Net has an encoder-decoder structure and was originally developed for medical image segmentation. The U-net architecture applied in this study was similar to that used in [26]; only the size of the input image was changed to 512 × 512. The AE system was built by removing the skip connections of the U-net. In this section, the humerus segmentation results of the proposed method with U-net and AE are visually compared.

**Figure 7:**
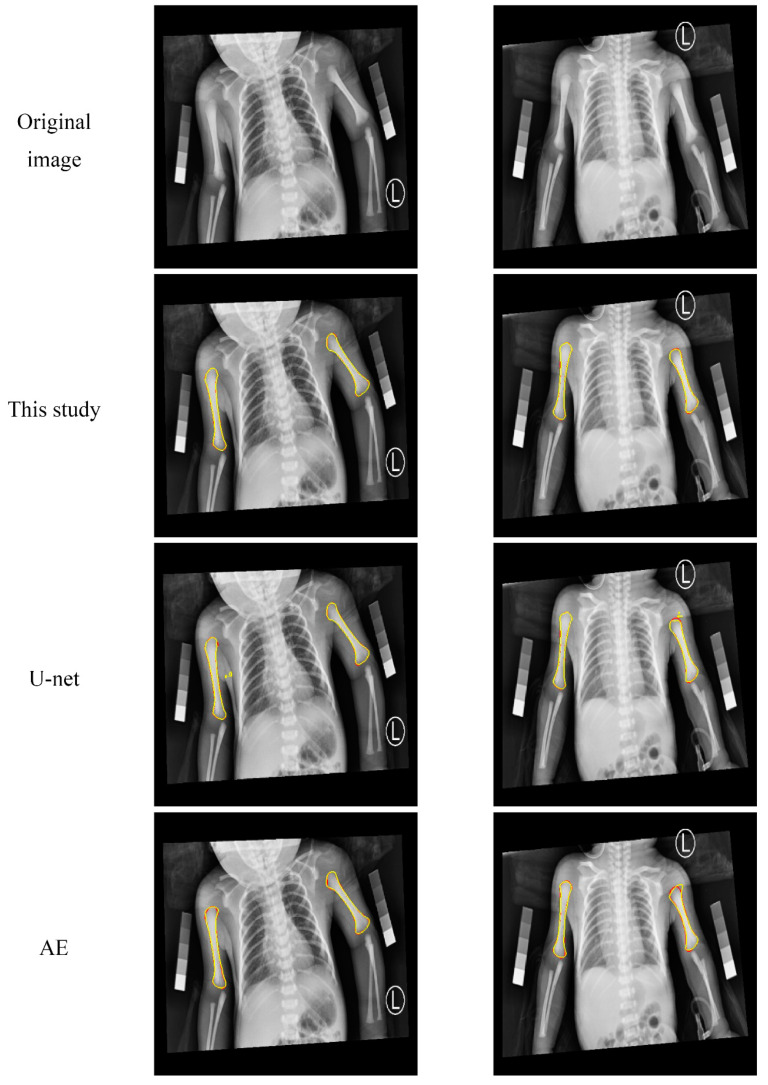
Visual comparison of the humerus segmentation results. The first row shows two examples of the original image. The second, third, and fourth rows represent the segmentation results of the proposed method, U-net, and auto-encoder (AE), respectively. The ground truth contours are in red, and segmented contours (in yellow) of the automatic method overlay on the ground truth contours.

Figure 7 shows two examples of the humerus segmentation results from a premature infant radiograph. The first row shows two examples of the original image. The second, third, and fourth rows demonstrate the segmentation results of the proposed method, U-net, and AE, respectively. The ground truth contours are in red, and segmented contours (in yellow) of the automatic method are overlaid on the ground truth contours. The segmented contours by the proposed method almost overlapped with the ground truth contours. Even in areas where the boundaries were not obvious, such as the upper and lower edges of the humerus, they could be segmented quite well. The U-net method also yielded good results, but there were some false positives generated, as shown in Figure 7. The results obtained by the AE method were slightly worse, and the contours were not as smooth and accurate as with the other two methods.

**Figure 8:**
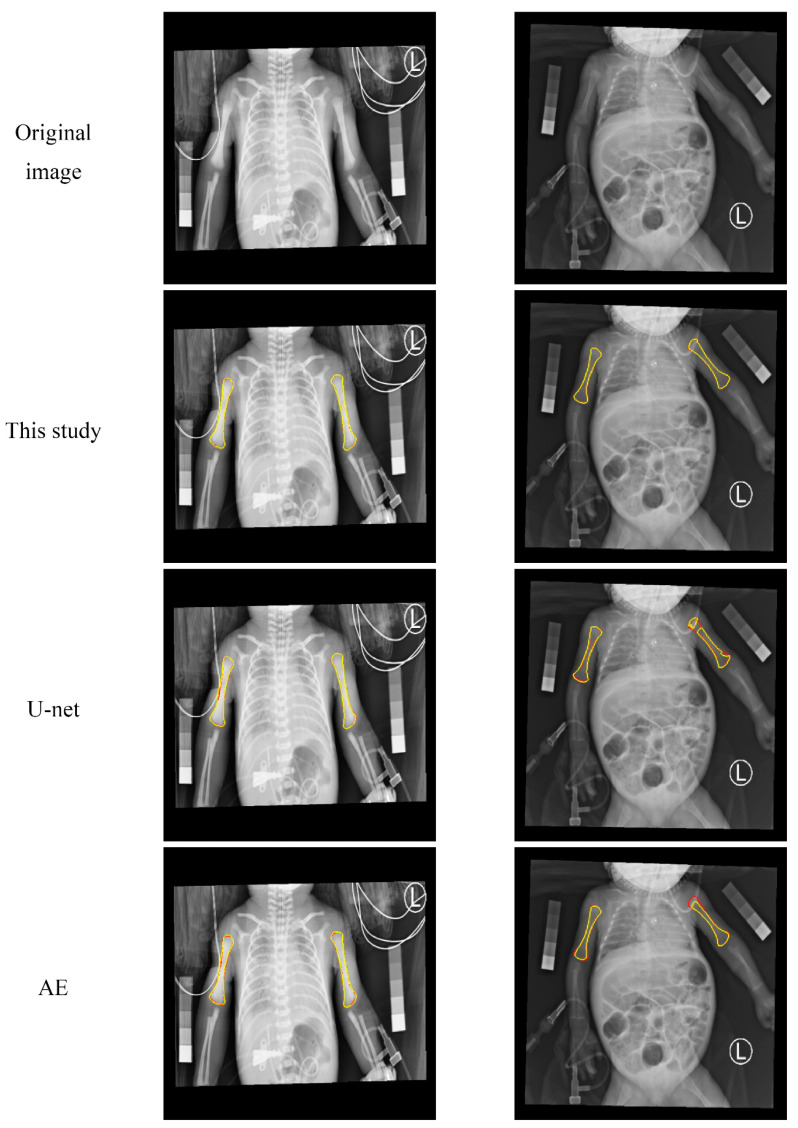
Visual comparison of the humerus segmentation results. The first row shows two examples of the original image. The second, third, and fourth rows represent the segmentation results of the proposed method, U-net, and AE, respectively. The ground truth contours are in red, and segmented contours (in yellow) of the automatic method overlay on the ground truth contours.

Figure 8 shows two other examples. In both, medical tubes appear on or near the humerus. The results show that the method of this study was not easily affected by medical tubes and was able to obtain good results. In contrast, the contours of the other two methods are easier to pull away by the medical tubes, resulting in poor segmentation results.

### 3.2. Quantitative Analysis of the Segmentation Results

For the quantitative analysis, the segmentation results were evaluated using the following five performance metrics: Dice similarity coefficient [27] (DSC), positive predictive value [19] (PPV), sensitivity [19] (SEN), mean absolute distance [28] (MAD), and Hausdorff distance [29] (HD). These evaluation metrics are defined below:

(4) $$DSC=\frac{2\left|A\cap B\right|}{\left|A\right|+\left|B\right|}$$

(5) $$PPV=\frac{\left|A\cap B\right|}{\left|B\right|}$$

(6) $$SEN=\frac{\left|A\cap B\right|}{\left|A\right|}$$

(7) $$MAD=\frac{1}{2}\left\{\frac{\sum_{a\in A_{c}}min_{b\in B_{c}}d\left(a,b\right)}{\left|A_{c}\right|}+\frac{\sum_{b\in B_{c}}min_{a\in A_{c}}d\left(b,a\right)}{\left|B_{c}\right|}\right\}$$

(8) $$HD=max\left\{max_{a\in A_{c}}(min_{b\in B_{c}}d\left(a,b)\right),\;max_{b\in B_{c}}(min_{a\in A_{c}}d\left(b,a)\right)\right\}$$

where *A* and *B* denote the humerus areas of the manual ground truth and the automatic segmentation, respectively. *A_c_* and *B_c_* refer to the contours of A and B, respectively. *a* denotes the pixel on the contour *A_c_*, while *b* denotes the pixel on the contour *B_c_*. *d*(*a*, *b*) represents the Euclidean distance between *a* and *b*. Among these five metrics, DSC, PPV, and SEN were area-based indicators. DSC measured the overlap degree between the manual and the automatic segmentations. PPV measured the ratio of correctly segmented area to the whole automatic segmentation area, while SEN measured the ratio of correctly segmented area to the whole manual segmentation area. On the other hand, MAD and HD were boundary-based metrics. MAD measured the average minimum distance between contour *A_c_* and *B_c_*. HD measured the maximum distance between contour *A_c_* and *B_c._* A smaller value of these two metrics indicated higher segmentation accuracy. Thus, an accurate segmentation would achieve a high value in area-based metrics and a low value in boundary-based metrics. Usually, area-based and distance-based indicators would be simultaneously considered, which made it easier to comprehensively evaluate the quality of the segmentation results.

The average evaluation value of all testing images was used to evaluate the performance of the system. A five-fold cross-validation process was employed. The dataset was randomly divided into five groups. Each group alternately served as the unseen testing image dataset while the remaining four groups merged as the training dataset. Augmentation of rotation with ±15 degrees was applied for the training dataset. The quantitative results are shown in Table 1. The DSC values were calculated with a per-patient basis on the 512 × 512 images. The proposed system successfully obtained a high DSC value of 97.81%, where nearly all of the left and right humerus could be successfully segmented under complicated image conditions. The performance of the proposed system was higher than that of the U-net. Both achieved high DSC values of over 97% and were better than the AE of 95.85%, which had no skip connection.

In the proposed system, the general setting of 3 × 3 was used to set the filter size of the convolution. The settings of growth rate and layer were randomly chosen at the beginning after finding out the combination that could make the system stable, then further analyzing the adjacent combination. Appendix A Table A2 lists some stable settings of growth rate r, and layer l in the dense block, including (r, l) = (5, 6), (6, 5), (6, 6), (6, 7), and (7, 6). The average DSC values obtained by the five settings were very close and as high as 97.66–97.81%. The chosen settings (6, 5) require the least training time and achieve the highest DSC value.

In addition to DSC, the three methods were compared in terms of PPV, SEN, MAD, and HD. The results (mean + standard deviation) are shown in Table 2. As the table shows, this study’s approach is also superior to the other methods in the other four indicators. The MAD and HD obtained by the proposed method were 0.12 mm and 1.11 mm, which were approximately equal to 0.26- and 2.41-pixel intervals, respectively. The results showed that the method proposed by this study had quite good segmentation results and accuracy. Moreover, observing the standard deviations of all indicators, the values of the proposed method were also smaller than the others, which indicated that the method proposed by this study had superior stability to interpatient variability.

### 3.3. BMD Estimation in Prematurity

A total of 210 premature infants were included in this experiment. On each premature infant radiograph, the BMDs were estimated in each defined ROIs, as mentioned in Section 2.4, including the upper, middle, and bottom parts of the humerus in the left and right upper arms. Table 3 shows the mean and the standard deviation of the estimated BMDs from premature infant radiographs. Due to the unstable vital signs of premature infants and the need for life support from incubators, the DEXA bone marrow density examination for prematurity was clinically scanty. There are no good diagnostic biochemical tests for rickets in preterm infants, and the serum calcium has only a weak correlation with the BMD. Previously, the scoring method of forearm radiography included grade 0 as normal, grade I as mineral rarefaction only, grade II as the change of grade I plus metaphyseal cupping and/or fraying of radius and/or ulna, and grade III as changes of grade II with fractures. According to Tsukahara’s study [26], the abovementioned scoring system corresponding to BMD values obtained by lumbar DEXA in prematurity reported as 0.245 ± 0.031 g/cm^2^ for grade 0, 0.197 ± 0.036 g/cm^2^ for grade I and 0.191 ± 0.033 g/cm^2^ for grade II, which are all between 0.1 and 0.3. The appendicular skeleton, such as the humerus, is largely composed of cortical bone, while the spine is mainly formed of trabecular bone. Therefore, the BMD of the humerus in this study’s estimated results was higher than the BMD from the lumbar spine.

## 4. Discussion

This study aimed to develop an alternative approach to DEXA testing for BMD estimation since critical infants cannot tolerate transportation to the diagnostic laboratory. Standard phantoms were used as mineral density standard to contrast the interested humerus bones. For clinical practice, a CNN-based method for humerus segmentation and automatic BMD calculation were also proposed. The humerus BMD values were successfully estimated from 210 infants’ images. However, two important points should be discussed further.

### 4.1. Number of Parameters and Computation Time

The number of parameters in the proposed segmentation system is only about 0.7–0.8% of the U-net or the AE as shown in Table 4, which reveals a considerable amount of memory saving in this study’s method. Since more concatenation operations were used in the dense block, it took more time to process the data in the proposed system. Although the system’s training time in this study was higher than the U-net, the segmentation results are also better. Looking at the testing time required for each image, the speed was actually not much different and was acceptable.

### 4.2. Feasibility of the Soft Tissue Correction Mechanism

**Figure 9:**
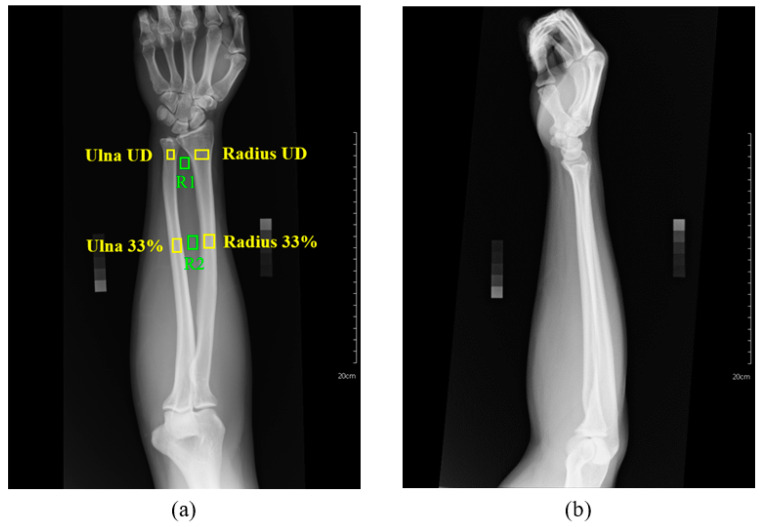
Different views of an adult forearm radiograph. (**a**) anterior-posterior view. Yellow rectangles represent different ROIs. Green rectangles represent the regions for estimating the average brightness of the soft tissues. (**b**) lateral view. Radius UD: ultra distal radius; Ulna UD: ultra distal ulna.

**Figure 10:**
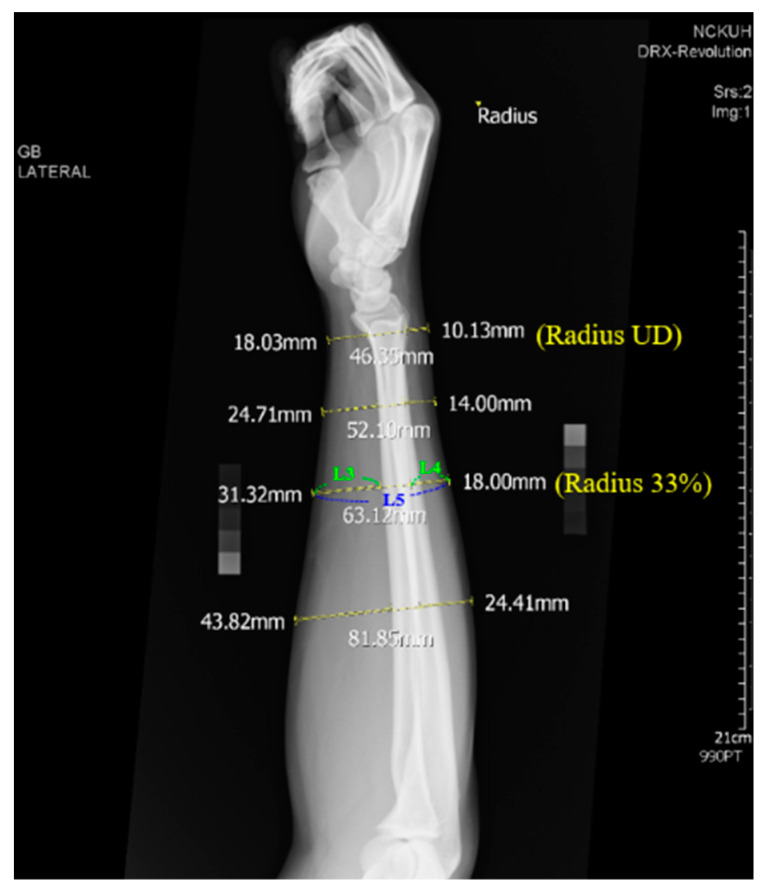
Materials for calculating the adjustment parameter S. For example, the parameter S for the ROI of Radius 33% is calculated by (L3 + L4)/L5, where these yellow dashed segments are drawn by an expert and the length of these segments are automatically calculated by the computer program. Radius UD: ultra distal radius.

DEXA usage is limited for premature infants, therefore adult radius and ulna were used to evaluate the feasibility of the soft tissue correction mechanism in this study’s technique for measuring BMD from the radiograph. The experiment was described as follows. The estimated BMDs at different ROIs, including radius 33%, ulna 33%, ultra distal radius(radius UD), and ultra distal ulna (ulna UD), of an adult forearm from the obtained linear regression equation are tested statistically for their correlation with the BMDs of the corresponding positions measured by DEXA. Ten subjects (five males and five females) were included in this experiment. The BMDs of each subject’s right forearm were measured by a Prodigy DEXA scanner (GE Lunar, Madison, WI, USA) and treated as the ground truth. Standard AP and LAT view radiograph of the right forearm, as illustrated in Figure 9, were obtained for estimating the BMD. A similar approach to Equation (2) was used to estimate the BMD at different ROIs. *I_y_* is the average brightness of the area to be estimated. *I_b_* is the average brightness of the region R1 (for ulna UD and radius UD) or R2 (for ulna 33% and radius 33%) in Figure 9a. The adjustment parameter S was calculated by dividing the thickness of the soft tissue by the thickness of the arm. For example, the parameter S for the ROI of Radius 33% is calculated by (L3 + L4)/L5, as illustrated in Figure 10, where the yellow dashed segments are drawn by an expert and the length of these segments are automatically calculated by the computer program.

**Figure 11:**
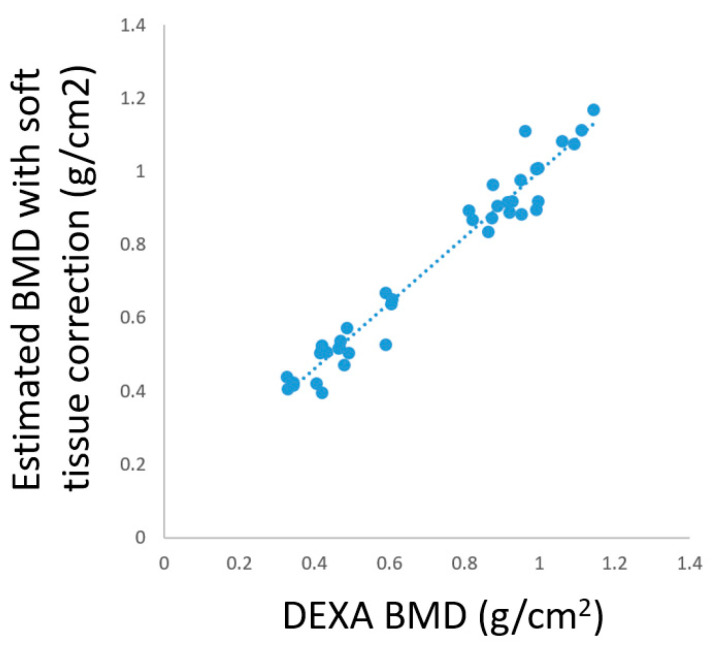
Correlation analysis between DEXA BMDs and estimated BMDs with soft tissue correction.

The results of estimated BMDs (with and without soft tissue correction) and DEXA BMDs are shown in Table 5. Through the soft tissue correction mechanism, the mean error could be effectively reduced from 0.416 to 0.051. Correlation analysis between DEXA BMDs and estimated BMDs with soft tissue correction is illustrated in Figure 11. There was a high correlation between the BMD obtained by DEXA and the estimated BMD with soft tissue correction, for which the correlation coefficient was 0.9799. This meant that the soft tissue correction mechanism adopted in this study was feasible and effective.

### 4.3. Limitation and Strength

This study had limitations. This study adopted standard and effective U-net related methods for segmentation, not including other published methods, such as UNet++ [30], that might further improve image segmentation accuracy. The segmentation result of this study was adequate for the subsequent BMD calculation with the proposed phantom calibration. The considerable number of 210 patients’ images with standard phantoms was the strength of this study. A significant trend was observed that the implemented new methods and ideas might impact clinical care.

## 5. Conclusions

This study provided a feasible alternative approach for the BMD assessment of critical infants by estimating the humerus mineral content from routine chest radiographs. The proposed CNN-based system for humerus segmentation is promising, and the end-to-end segmentation strategy is convenient for practical applications. The proposed architecture also achieves good accuracy and great network parameter savings. The estimated BMD values, after correction for soft tissue with standard phantoms, are close to those in previous studies. The study’s preliminary results might accelerate related research on BMD assessment from routine chest radiographs and might make early detection of BMD loss in critical infants, even adult patients.

## Figures and Tables

**Table 1:** Dice similarity coefficient DSC (%) of the proposed system compared with other convolutional neural network- (CNN)-based methods. A five-fold cross-validation process is employed. Each fold (*k*) alternately serves as the unseen testing dataset. (mean ± standard deviation). The auto-encoder (AE) system was built by removing the skip connections of the U-net.

	*k* = 1	*k* = 2	*k* = 3	*k* = 4	*k* = 5	Avg.
This study	97.65 ± 1.36	97.82 ± 1.71	97.97 ± 0.71	97.90 ± 0.94	97.68 ± 0.98	97.81 ± 1.14
U-net [26]	96.51 ± 3.04	97.39 ± 2.54	97.87 ± 0.66	97.25 ± 1.67	97.10 ± 1.58	97.23 ± 1.90
AE	95.46 ± 1.88	96.28 ± 1.45	96.60 ± 0.64	95.78 ± 1.78	95.14 ± 3.00	95.85 ± 1.75

**Table 2:** Quantitative comparison of the segmented results of the proposed system, U-net, and AE in terms of DSC, positive predictive value (PPV), sensitivity (SEN), mean absolute distance (MAD), and Hausdorff distance (HD). (mean ± standard deviation). The AE system was built by removing the skip connections of the U-net.

	DSC (%)	PPV (%)	SEN (%)	MAD (mm)	HD (mm)
This study	97.81 ± 1.14	97.84 ± 1.27	97.79 ± 1.41	0.12 ± 0.06	1.11 ± 1.24
U-net [26]	97.23 ± 1.90	97.42 ± 2.08	97.06 ± 2.20	0.15 ± 0.10	1.51 ± 1.96
AE	95.85 ± 1.75	95.81 ± 2.38	95.97 ± 2.24	0.23 ± 0.11	1.57 ± 1.78

**Table 3:** Estimated BMDs from premature infant radiographs. The regions of interest (ROIs) include the upper, middle, and bottom parts of the humerus in the left and right upper arms (in g/cm^2^).

	Left Upper Arm			Right Upper Arm		
	Upper	Middle	Bottom	Upper	Middle	Bottom
Mean	0.32	0.37	0.32	0.32	0.36	0.31
S.D.	0.06	0.06	0.09	0.06	0.07	0.09

**Table 4:** Number of parameters, training time, and testing time used in the proposed system compared with other CNN-based methods.

	This Study	U-net	AE
Number of parameters	258,055	31,041,409	33,826,689
Training time (h/fold)	6.8	4.2	21.5
Testing time per image (s)	0.30	0.23	0.23

**Table 5:** Comparison between estimated BMDs and DEXA BMDs (in g/cm^2^).

Case	Region	(1) Estimated BMD without Soft Tissue Correction	(2) Estimated BMD with Soft Tissue Correction	(3) DEXA BMD	Difference between (1) and (3)	Difference between (2) and (3)
Male 1	Radius 33%	1.662	1.083	1.061	0.601	0.022
	Ulna 33%	1.732	1.169	1.145	0.587	0.024
	Radius UD	1.079	0.649	0.607	0.472	0.042
	Ulna UD	0.945	0.535	0.471	0.474	0.064
Male 2	Radius 33%	1.532	1.111	1.114	0.418	0.003
	Ulna 33%	1.515	1.075	1.094	0.421	0.019
	Radius UD	0.978	0.637	0.605	0.373	0.032
	Ulna UD	0.871	0.504	0.492	0.379	0.012
Male 3	Radius 33%	1.393	0.976	0.950	0.443	0.026
	Ulna 33%	1.532	1.111	0.962	0.570	0.149
	Radius UD	0.956	0.572	0.488	0.468	0.084
	Ulna UD	0.876	0.504	0.417	0.459	0.087
Male 4	Radius 33%	1.400	0.964	0.877	0.523	0.087
	Ulna 33%	1.310	0.868	0.823	0.487	0.045
	Radius UD	0.802	0.522	0.420	0.382	0.102
	Ulna UD	0.765	0.424	0.345	0.420	0.079
Male 5	Radius 33%	1.682	1.008	0.997	0.685	0.011
	Ulna 33%	1.669	1.006	0.992	0.677	0.014
	Radius UD	1.155	0.667	0.592	0.563	0.075
	Ulna UD	0.987	0.525	0.422	0.565	0.103
Female 1	Radius 33%	1.156	0.889	0.922	0.234	0.033
	Ulna 33%	1.149	0.873	0.874	0.275	0.001
	Radius UD	0.764	0.519	0.467	0.297	0.052
	Ulna UD	0.687	0.439	0.327	0.360	0.112
Female 2	Radius 33%	1.241	0.917	0.927	0.314	0.010
	Ulna 33%	1.216	0.892	0.812	0.404	0.080
	Radius UD	0.841	0.506	0.434	0.407	0.072
	Ulna UD	0.742	0.416	0.344	0.398	0.072
Female 3	Radius 33%	1.226	0.835	0.864	0.362	0.029
	Ulna 33%	1.276	0.882	0.952	0.324	0.070
	Radius UD	0.753	0.471	0.479	0.274	0.008
	Ulna UD	0.698	0.421	0.406	0.292	0.015
Female 4	Radius 33%	1.294	0.905	0.890	0.404	0.015
	Ulna 33%	1.306	0.914	0.916	0.390	0.002
	Radius UD	0.753	0.515	0.466	0.287	0.049
	Ulna UD	0.702	0.404	0.331	0.371	0.073
Female 5	Radius 33%	1.362	0.917	0.997	0.365	0.080
	Ulna 33%	1.365	0.895	0.992	0.373	0.097
	Radius UD	0.853	0.527	0.592	0.261	0.065
	Ulna UD	0.722	0.396	0.422	0.300	0.026
				Mean:	0.416	0.051

Radius UD: ultra distal radius; Ulna UD: ultra distal ulna.

## Appendix A

**Table A1:** Parameter settings of the proposed network.

Parameter	Related Settings
Filter size of convolution in the main network architecture	3 × 3 or 1 × 1, as shown in Figure 2
Filter size of max pooling in the main network architecture	2 × 2
Batch size	4
Epoch	100
Learning rate	0.001, with a decay of 0.5% after each epoch
Optimizer	Adam
Loss function	L2-norm, as Equation (1)
Layer (l) in the dense block	5
Growth rate (r) in the dense block	6
Filter size of convolution in the dense block	3 × 3

**Table A2:** DSC (%) of the proposed system with different settings of growth rate (r) and layer (l) in the dense block. A five-fold cross-validation process was employed.

Setting	r	l	DSC Avg. (%)	Training Time (hour/fold)
1	5	6	97.66 ± 1.40	7.8
2	6	5	97.81 ± 1.14	6.8
3	6	6	97.79 ± 1.18	8.8
4	6	7	97.79 ± 1.28	10.9
5	7	6	97.81 ± 1.11	9.9

DSV AVG. Average of Dice similarity coefficient value (mean ± standard deviation).
